# Predictors of Failure from Primary Therapy for Giardiasis in San Diego: A Single Institution Retrospective Review

**DOI:** 10.3390/pathogens8040165

**Published:** 2019-09-27

**Authors:** Anjan Debnath, Sharon L. Reed, Sheldon R. Morris

**Affiliations:** 1 Center for Discovery and Innovation in Parasitic Diseases, Skaggs School of Pharmacy and Pharmaceutical Sciences, University of California San Diego, La Jolla, CA 92093, USA; adebnath@ucsd.edu; 2 Department of Pathology, University of California San Diego, La Jolla, CA 92093, USA; slreed@ucsd.edu; 3 Department of Medicine, University of California San Diego, La Jolla, CA 92093, USA; 4 Departments of Medicine and Family Medicine and Public Health, University of California San Diego School of Medicine, San Diego, CA 92103, USA

**Keywords:** metronidazole, giardia, HIV, treatment failure, retrospective

## Abstract

This study aimed to determine the presence of giardiasis among HIV patients in San Diego, the rate of failure of metronidazole treatment, and factors associated with treatment failure. We used a 7 year retrospective single-center case series of HIV-infected individuals with giardiasis at University of California San Diego Medical Center. Data were analyzed for the changes in the hematological, biochemical, and immunologic results at pre- and at-diagnosis levels. We also compared the changes at the diagnosis level among patients who were treated successfully and those who experienced treatment failure as defined by retreatment with a second course of antibiotics. In 29 *Giardia lamblia*-infected HIV patients, following diagnosis of *G. lamblia*, there was a non-significant decrement in cluster of differentiation 4 (CD4), but a statistically significant increase in the number of white blood cell (WBC). Other indices did not differ between pre- and at-diagnosis levels. Twenty patients (69%) were treated with a single course of metronidazole or tinidazole and seven patients (24.1%) were treated with more than one course of metronidazole. These seven patients had statistically significant higher hemoglobin at the time of diagnosis, but further studies are required to confirm if this is a consistent finding and if this can predict failure from primary therapy.

## 1. Introduction

Almost two hundred million cases of giardiasis are reported each year worldwide [1] with an estimated 1.2 million in the US [2]. In developed countries, *Giardia lamblia*, the protozoan responsible for giardiasis, infects about 2% of adults and approximately 6–8% of children [3,4]. The prevalence of giardiasis is higher in developing countries [5]. According to the WHO Foodborne Disease Burden Epidemiology Reference Group (FERG), giardiasis produced 171,100 disability-adjusted life years (DALYs) in 2010 [1]. In the US, human giardiasis is the most common intestinal parasitic disease and the single most frequently identified pathogen in all drinking water outbreaks from 1971 to 2006 with 121 outbreaks [4]. In 2010, the total number of reported cases of giardiasis in the US was 19,927 [4]. Each year, hospitalizations resulting from giardiasis cost approximately $34 million; additionally, each ambulatory care visit for giardiasis costs $121–$273 [5]. While the national incidence rate of giardiasis in 2016 was 6.4 cases per 100,000 persons, California reported the incidence rate of 7 cases per 100,000 persons. On the other hand, San Diego County’s incidence rate was 12.1 in 2016 [6]. The high rate in San Diego may be partially explained by a larger refugee population since about 30% of giardiasis cases were reported from refugees, and the median age of these cases was 10 years [6]. Among non-refugee cases, the median age was 37 years, and they were reported from travelers, campers, and recreational water users but the number of HIV-infected patients was not reported [6]. There was an imbalance by sex with 64% of giardiasis cases from San Diego were in males [6].

Because of its low infectious dose and potential for causing food and water outbreaks in the United States, the National Institutes of Health (NIH) has listed *G. lamblia* as a category B priority biodefense pathogen. In 2004, *Giardia* was included in the WHO Neglected Diseases Initiative due to its link with poverty [7].

Metronidazole is the most common drug used to treat giardiasis [8]. It is cheap and has been in use for more than 50 years, but it has several adverse effects, such as nausea, headache, vertigo, vomiting, diarrhea, or constipation [9]. It is recommended to avoid alcohol consumption while taking this drug due to the inhibition of aldehyde dehydrogenase. Long-term use of this drug may cause chromosomal aberrations in Crohn’s disease [10] and occasionally peripheral neuropathy [11]. Standard treatment with metronidazole requires 250 mg three times daily dosing for 5–7 days for giardiasis [12]. Newer metronidazole derivatives such as tinidazole [13] and nitazoxanide, a nitrothiazolyl-salicylamide derivative [14], have fewer side-effects and shorter treatment courses. Other drugs, such as furazolidone, albendazole, and paromomycin, are used for giardiasis to a lesser extent with similar and/or lower success rates. Despite the efficacy of nitroimidazole drugs, treatment failures in giardiasis have been reported in up to 40% of cases [15].

Clinical resistance of *G. lamblia* to metronidazole is proven and cross resistance occurs with the newer drugs, tinidazole and nitazoxanide [16], so drug resistance is a concern with all commonly used antigiardial drugs [16,17]. Studies on prevalence of metronidazole treatment failure for giardiasis are scarce in the USA.

Immunocompromised patients are also one of the high-risk groups for *G. lamblia* infection. Earlier studies showed that individuals with HIV/AIDS were more prone to *G. lamblia* infection [18,19,20,21]. Considering the relative paucity of data on giardiasis and treatment failure among giardiasis patients with HIV in the US, we carried out a single institution retrospective case series review study among *G. lamblia*-infected HIV patients to determine the associated risk factors and response to primary therapy.

## 2. Results

### 2.1. Characteristics of the Study Population

We identified 29 HIV-infected patients positive for *G. lamblia*. All were adult males with an average age of 43.17 ± 9.85 years and weight of 179.86 ± 26.91 lbs, as shown in Table 1. Among 29 patients, 16 were White (55.2%), 11 were Hispanic (37.9%), 1 African American (3.4%), and 1 Asian Pacific Islander (3.4%). Though the travel history of all patients was not always available, one patient reported exposure to dirty hurricane water while traveling to the east coast. Two patients had a travel history of visiting Mexico. All patients were on antiretroviral treatment at time of the diagnosis.

### 2.2. Clinical Status of Patients

**Figure 1:**
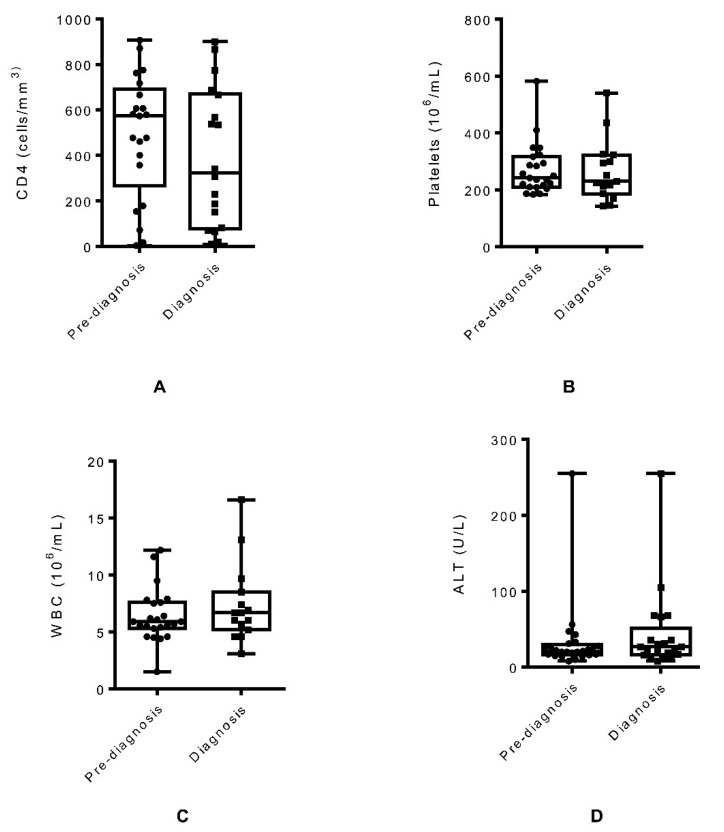

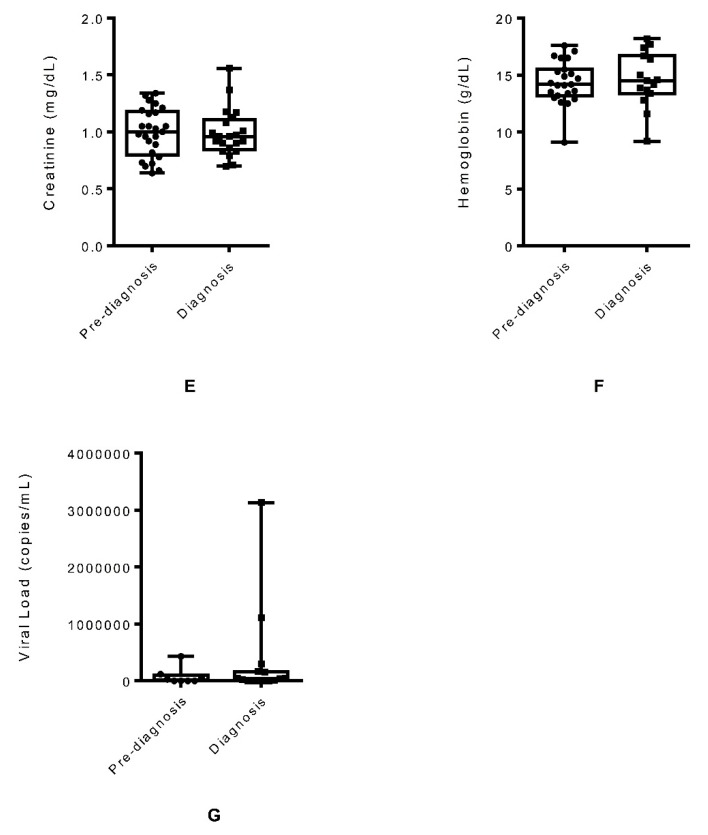
Changes in cluster of differentiation 4 (CD4) (**A**), platelets (**B**), white blood cell (WBC) (**C**), alanine aminotransferase (ALT) (**D**), creatinine (**E**), hemoglobin (**F**), and HIV viral load (**G**) associated with giardiasis at the pre-diagnosis and at-diagnosis levels.

For 11 patients (37.9%), both pre-diagnosis and at-diagnosis data were available. Following diagnosis of *G. lamblia* infection, we found a non-statistically significant decrease in cluster of differentiation 4 (CD4) (mean 61.38 cells/mm^3^ decrease, 95% CI: −25.79, 148.6, p = 0.15) and platelets (mean 13.6 × 10^6^/mL decrease, 95% CI: −48.38, 75.58, p = 0.63) in patients compared to the values obtained before diagnosis of the parasitic infection, as shown in Figure 1A,B. There was a statistically significant increase in the number of white blood cell (WBC) in *G. lamblia*-infected HIV patients at the time of diagnosis compared to the WBC count before diagnosis of giardiasis (mean 2.47 × 10^6^/mL increase, 95% CI: −4.116, −0.8236, p = 0.008), as shown in Figure 1C. For other clinical data, the values reported at the time of diagnosis were similar to the pre-diagnostic values. There were non-statistically significant changes in alanine aminotransferase (ALT) (mean 3.765 U/L increase, 95% CI: −15.69, 8.161, p = 0.51), creatinine (mean 0.046 mg/dL increase, 95% CI: −0.139, 0.046, p = 0.3), and hemoglobin (mean 0.23 g/dL increase, 95% CI: −1.62, 1.162, p = 0.72) in HIV patients between pre-diagnosis and at the time of diagnosis of *G. lamblia* infection, as shown in Figure 1D–F. HIV viral load for pre-diagnosis and at the time of diagnosis was available for only six patients. When compared, there were no statistically significant changes in the viral load before diagnosis and at the time of diagnosis of *G. lamblia* (mean 752,808 copies/mL increase, 95% CI: −1,996,000, 490,041, p = 0.18), as shown in Figure 1G.

### 2.3. Association of Giardiasis with Symptomatology

When we studied the association of *G. lamblia* infection with symptoms in HIV-infected patients, we observed diarrhea, epigastric pain, and weight loss as the most frequent symptoms. Other symptoms included bloating, dyspepsia, and vomiting. While most patients (28 out of 29, 96.6%) experienced diarrhea, 16 patients (55.2%) reported epigastric pain, and 15 patients (51.7%) showed the symptom of weight loss. Bloating, dyspepsia, and vomiting had very low frequency with 6.9%, 10.3%, and 13.8%, respectively, as shown in Table 2.

### 2.4. Primary Treatment of Giardiasis in HIV Patients

Most of the patients (25 patients, 86.2%) were primarily treated with metronidazole. Two patients (6.9%) were treated with tinidazole and two patients were given ciprofloxacin (for concomitant *Shigella*), as shown in Table 3. The dosing regimen for metronidazole was either 250 mg three times a day (TID) or 500 mg TID or 500 mg two times a day (BID). The duration of treatment with metronidazole varied between 5 days to 14 days. Two patients received 2 g once a day (QD) tinidazole; one patient was treated with tinidazole for 7 days and another patient for one day. The patients who received 500 mg BID ciprofloxacin also had *Shigella* infection, but no metronidazole was found in the electronic medical record medication list. Only one patient reported an adverse effect due to metronidazole, with nausea in the absence of drinking alcohol. This patient was switched to nitazoxanide at 500 mg BID for 3 days.

Two patients who were treated successfully with primary therapy had an underlying condition of gastroesophageal reflux disease (GERD) and one patient had *Helicobacter pylori* infection. Another patient had an exposure to tuberculosis (TB) and was also positive for syphilis.

### 2.5. Treatment of Giardiasis with More Than One Course of Metronidazole

Out of 29 *G. lamblia*-positive HIV patients, 15 had follow up stool testing for the presence of *G. lamblia* trophozoites or cysts and 14 patients did not. Seven patients (24.1%) were treated with more than one course of metronidazole. The treatment courses and diagnostic testing of these seven patients are provided in Table 4.

Several patients had comorbidities, with patient # 1 having overlapping conditions of syphilis, shingles, and *Shigella* infection. Patient # 2 and 3 were suffering from hepatitis B. Patient # 3 also experienced GERD. Patient # 6 had underlying conditions of syphilis and meningioma.

### 2.6. Analysis of Lab Data to Identify Predictors of Multiple Courses of Metronidazole Treatment

**Figure 2:**
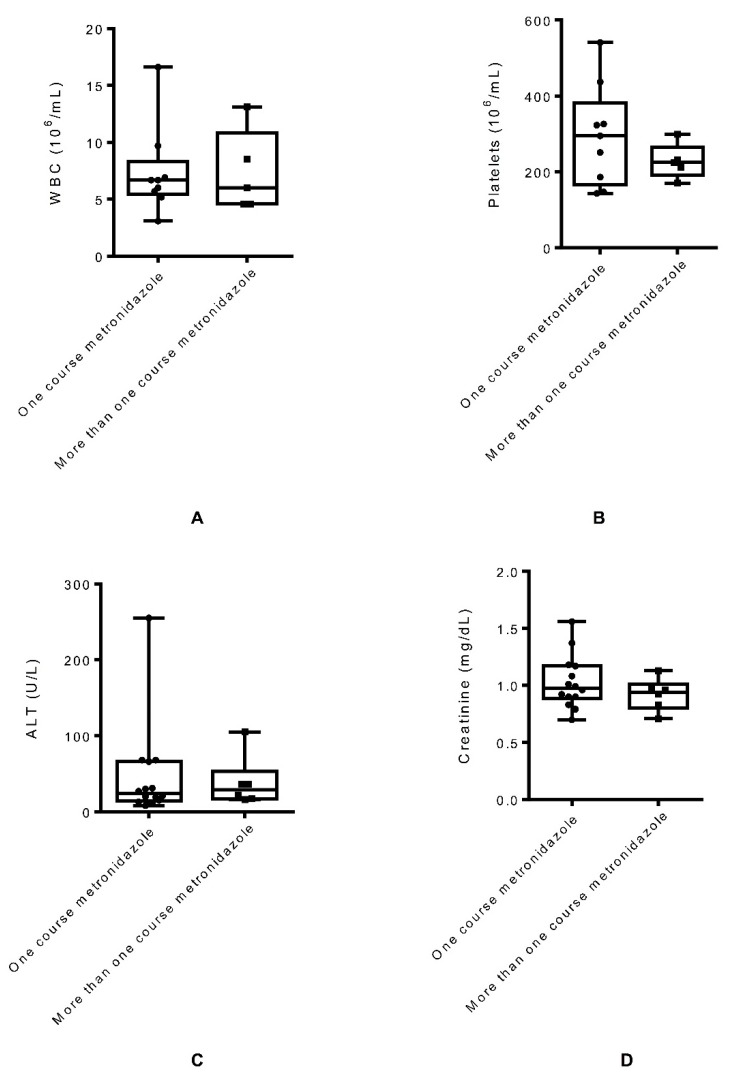

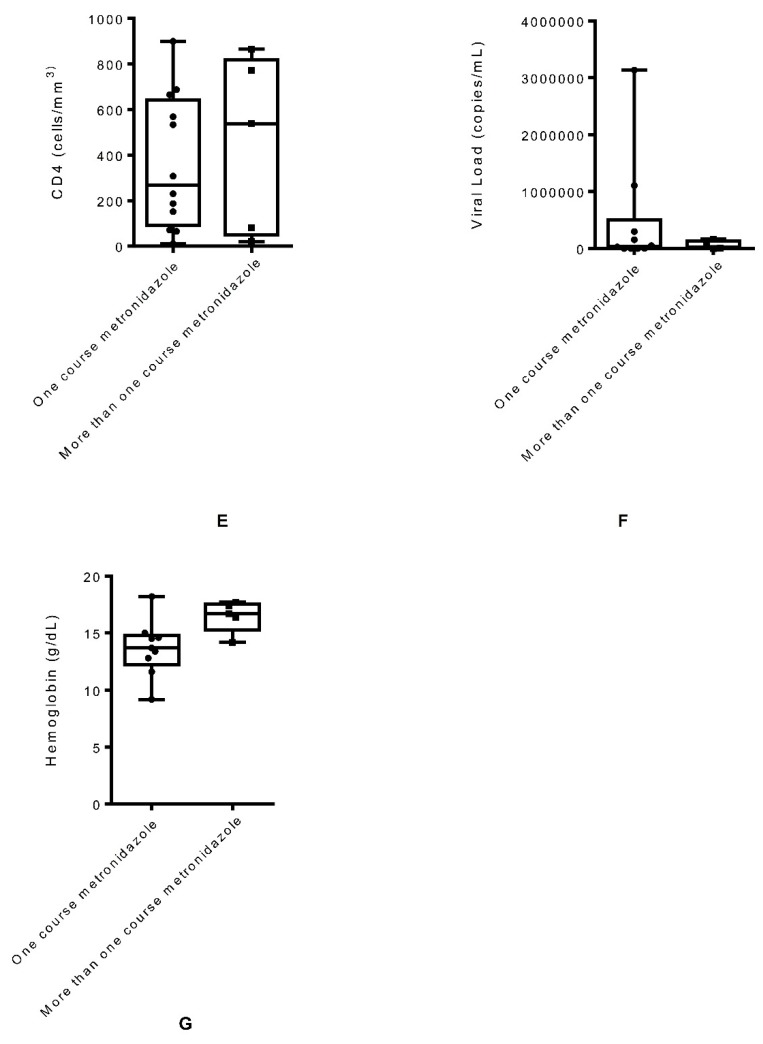
Comparison of WBC (**A**), platelets (**B**), ALT (**C**), creatinine (**D**), CD4 (**E**), HIV viral load (**F**), and hemoglobin (**G**) at the diagnosis level between giardiasis patients who required a single course of metronidazole treatment and those who were treated with multiple courses of metronidazole.

Since clinical data were compared only at-diagnosis level, unpaired sample T tests were performed to compare at-diagnosis hematological, biochemical, and immunologic clinical parameters of the patients who required more than one course of metronidazole for the treatment of giardiasis and those who required a single course of metronidazole treatment. When WBC count was compared between patients who required a single course of metronidazole treatment and those who required more than one course of metronidazole, the difference was not statistically significant (p = 0.99), as shown in Figure 2A. Similarly, non-statistically significant differences were found with platelets (p = 0.3), as shown in Figure 2B; ALT (p = 0.78), as shown in Figure 2C; creatinine (p = 0.32), as shown in Figure 2D; CD4 (p = 0.6), as shown in Figure 2E; and HIV viral load (p = 0.4), as shown in Figure 2F. When the hemoglobin level at the diagnosis of *G. lamblia* between patients who were treated with a single course of metronidazole treatment and those who required more than one course of metronidazole was compared, there was a statistically significant increase in the hemoglobin (p = 0.04) in the patients who required multiple courses of metronidazole treatment, as shown in Figure 2G.

## 3. Discussion

Though some studies were conducted earlier to assess the prevalence of *G. lamblia* in HIV patients, very few were reported from the US. Our study aimed to determine the treatment regimen(s) followed in giardiasis in HIV-infected patients and the treatment failure rates. Treatment failures of giardiasis using metronidazole has been reported in up to 40% of cases in patients referred to the Hospital of Tropical Diseases in London [15]. In a retrospective study, 22% of Spanish travelers who returned from Asian countries showed refractory giardiasis [22]. Similarly, in another study in Spain, where patients with chronic giardiasis were prospectively analyzed, about 20% were refractory to the treatment with metronidazole or tinidazole [23,24]. Some potential factors for treatment failures may be reinfection, immunosuppression, resistance to the drug, insufficient drug levels, sequestration in the gallbladder or pancreatic ducts, or co-infection with or selection of other microorganisms in the gut microbiota [23,25]. Unfortunately, there is no easy test for resistance and isolation of the strain and axenization for sterile growth are required before resistance testing can be performed. In this 7 year retrospective single-center chart review of giardiasis cases in HIV patients at University of California San Diego Health, we found that most patients did not receive definitive tests of cure, so our definition of possible failure was if patients received multiple courses of therapy. We reviewed different clinical parameters to determine the predictors of failure from primary therapy.

We compared different hematological, biochemical, and immunological clinical data before diagnosis and at the time of diagnosis of giardiasis in all 29 HIV patients to identify the factors associated with giardiasis in HIV patients. We found a statistically significant increase of WBC count at the diagnosis of giardiasis compared to the count available three to six months before diagnosis. Although the WBC count is not consistently increased with giardiasis, our finding of increased WBC requires further investigation to understand if it represents the impact of *Giardia* on patients that are immunosuppressed, or if the increase was due to co-infection with other organisms. We also observed decreased CD4 count in HIV patients who were diagnosed with *G. lamblia* infection, but this association was not statistically significant. An earlier study also found that low CD4 counts were linked to increased *G. lamblia* assemblage B replication in HIV patients [20]. When we evaluated the association of giardiasis and the symptomatology in HIV patients, diarrhea was the most frequent clinical manifestation. HIV patients with weakened immune systems are prone to gastrointestinal infections and often present diarrhea for which giardia should be considered in the differential diagnosis.

Twenty (70.0%) patients were treated successfully with a single course of primary metronidazole or tinidazole therapy. There was no consistent dose, frequency, and length of treatment used, suggesting an educational opportunity to establish guidelines on giardia management in HIV.

Seven (24%) patients required more than one course of treatment. These seven patients were investigated further as potential failures of primary therapy. One patient received five courses of treatment. As a limitation for our study, it was difficult to identify the true failure rate as the majority of the retreatment decisions (in 71% of the 7 patients) was not based on laboratory identification of persistent infection but continued symptoms alone. In at least one case, retreatment was based on a repeat positive multiplex PCR test, which should not be used as a test of cure, as *Giardia* DNA may be detected long after resolution of infection. Again, clear guidance on repeat testing after therapy may be needed. Almost a quarter of patients received repeat treatment, which is not different from non-HIV-infected individuals. These findings support the need for alternative treatment regimens for giardia. Refractory giardiasis patients were successfully treated with metronidazole–quinacrine combination therapy in one study [25]. In two other studies, patients with metronidazole-refractory giardiasis responded better with a combination of albendazole and metronidazole than monotherapy [26,27]. Although the dosing, duration, and adverse effects of this combination therapy are limited, future treatment with combinations of drugs should be studied as second line treatment.

To determine the factors responsible for failure in primary therapy, we compared the lab data available at the time of diagnosis between patients who were treated successfully with one round of primary therapy and those who required multiple courses of primary therapy. Though the differences in several clinical parameters at the diagnosis level between these two groups of patients were not statistically significant, there was a statistically significant increase in hemoglobin in the patients who required more than one course of metronidazole treatment compared to the patients successfully treated with one round of metronidazole. The reason for increased hemoglobin in the patients as a predictor of failure from primary therapy is unclear but may need further investigation.

## 4. Materials and Methods

### 4.1. Overview

We conducted a 7 year retrospective single-center chart review to determine the prevalence of metronidazole treatment failure for giardiasis among patients with HIV/AIDS and also to determine the associated risk factors.

### 4.2. Patient Selection

Subjects who visited the largest HIV clinic, the University of California San Diego (UCSD) Owen Clinic, between January 2011 and February 2018 were selected by having a positive test for *G. lamblia* by any method. Chart records were then examined to confirm eligibility, then abstracted for demographics, weight, recent travel, date of diagnosis, symptoms including diarrhea, weight loss, nausea or bloating, dyspepsia, vomiting, epigastric pain, any treatment of *Giardia*, dosing regimen and duration of treatment, concomitant infections, or underlying medical conditions. Records of physical examinations focusing on the abdomen and initial laboratory testing with stools for ova and parasite exams, results of a multiplex stool pathogen PCR (offered after September 2014), and stool cultures for enteropathogens including *Shigella, Salmonella, Campylobacter,* and enterotoxigenic *Escherichia coli* were collected. Stool PCR for identification of *Giardia* was performed using a Biofire FilmArray Gastrointestinal (GI) Panel from bioMerieux.

The hematological, biochemical, and immunologic results were categorized into pre-diagnosis (performed with 3–6 months prior to the positive result) and at-diagnosis (performed within two weeks of the positive result). Pre- and at-diagnosis platelets count, WBC, hemoglobin, alanine aminotransferase (ALT), creatinine, viral load, and CD4 count were recorded. The platelets, WBC, and hemoglobin were measured using the XN Automated System (Sysmex, Lincolnshire, IL, USA); ALT and creatinine were determined using a cobas c700 system (Roche, Indianapolis, IN, USA); CD4 was counted using a FACSCanto (Becton Dickinson, San Jose, CA, USA) and HIV viral loads were quantified using Roche HIV-1 quantitative real-time PCR run on a cobas 6800 System (Roche, Indianapolis, IN, USA).

Treatment failure was defined both by lab confirmation of presence of *Giardia* in at least one of three stool samples following therapy, and by symptoms, defined as persistence of diarrhea, bloating, abdominal pain, or weight loss after one or more courses of metronidazole treatment. Treatment success was defined as symptom resolution and absence of *Giardia* in three stool samples.

### 4.3. List of Inclusion Criteria

All patients met all of the following inclusion criteria. They were eligible if:

- >18 years old
- HIV infected
- Giardiasis case definition by the following criteria:
  - *Giardia* cysts or trophozoites detected in stool by microscopic examination of wet mounts
  - OR *Giardia* cysts or trophozoites identified by trichrome smear of concentrated stools
  - OR positive stool PCR for *Giardia*

### 4.4. Data Collection

The following data were collected from the medical records:

- Verify inclusion criteria at the time of diagnosis of *Giardia*
- Demographic data at the time of diagnosis of *Giardia*
- Travel history at the time of diagnosis and up to 90 days after diagnosis
- Clinical data at the time of diagnosis and up to 90 days after diagnosis
- Physical exam at the time of diagnosis and up to 90 days after diagnosis
- Lab data including microbiology, CD4, HIV viral load at the time of diagnosis and up to 90 days after diagnosis
- Medications including antibiotics, antiretroviral therapy (ART) at the time of diagnosis and up to 90 days after diagnosis

Eligible patients were identified by having a positive *Giardia* testing with follow-up at the HIV clinic from January 2011 to February 2018 with clinical details from the electronic medical record (Institutional Review Board (IRB) approval (Project #180699XL, 4/26/18)).

### 4.5. Statistical Methods

The data entry was performed using Excel and analyzed using GraphPad Prism. Exploratory analysis of the categorical variables was performed using percentages, and continuous variables were presented as mean ± standard deviation. Clinical data at pre- and at-diagnosis levels were compared using paired sample T tests with p < 0.05 as the level of statistical significance. At-diagnosis clinical data of the patients who required more than one course of metronidazole for the treatment of giardiasis and those who required a single course of metronidazole treatment were compared using unpaired sample T tests. The comparison of the clinical data was visualized using box and whisker plots.

### 4.6. Compliance with Ethics Guidelines

The study was approved by the Institutional Review Board (IRB) of University of California San Diego (UCSD) Human Research Protections Program (reference number: Project #180699XL, 4/26/18). For this type of study, formal consent is not required.

## 5. Conclusions

In conclusion, in this case series study of giardiasis among HIV-infected individuals, we found that clinical practice varied in diagnostic methods, treatment, and management. We observed the failure rate for primary treatment of 24%, which is not different from non-HIV-infected individuals. Our results support the development of giardia management guidance for HIV physicians and study of alternative treatments.

## Figures and Tables

**Table 1:** Characteristics of the HIV patients infected with *Giardia lamblia.*

Characteristics	Overall (n = 29)		
	No.	%	Average
*Gender*			
Male	29	100	
*Age (Years)*	-	-	43.17 ± 9.85
*Weight (lb)*	-	-	179.86 ± 26.91
*Race/Ethnicity*			
White	16	55.2	-
Hispanic	11	37.9	-
African American	1	3.4	-
Asian Pacific Islander	1	3.4	-
*ART* (*Antiretroviral Therapy*)			
Yes	29	100	-

**Table 2:** Clinical symptoms associated with giardiasis in HIV patients.

Characteristics	Overall (n = 29)	
	No.	%
*Clinical Symptoms*		
Diarrhea	28	96.6
Epigastric Pain	16	55.2
Weight Loss	15	51.7
Bloating	2	6.9
Dyspepsia	3	10.3
Vomiting	4	13.8

**Table 3:** Primary treatment of giardiasis in HIV patients.

Drugs	Overall (n = 29)		Dosing Regimen	Duration
	No.	%		
Metronidazole	25	86.2	250 mg three times a day (TID), 500 mg two times a day (BID), 500 mg TID	5–14 days
Tinidazole	2	6.9	2 g once a day (QD)	1 day, 7 days
Nitazoxanide (Switched from Metronidazole)	1	3.4	500 mg BID	3 days

**Table 4:** Treatment of giardiasis in HIV patients with more than one course of drug.

Patient #	Drugs	Dosing Regimen	Duration	Treatment	Follow-Up Testing
1	Metronidazole	250 mg TID	5 days	After diagnosis	
		500 mg TID	5 days	23 days after first course of treatment for diarrhea	11 months—Stool ova and parasite (O&P) negative
2	Metronidazole	500 mg TID	5 days	After diagnosis	
		500 mg TID	5 days	14 days after first course of treatment for diarrhea	N/A
		500 mg TID	15 days	7 days after second course of treatment	Stool O&P negative
3	Metronidazole	500 mg TID	14 days	After diagnosis	
		500 mg TID	10 days	3 months after first course of treatment for diarrhea	Stool O&P negative
4	Metronidazole	500 mg TID	7 days	After diagnosis	
		500 mg BID	7 days	One year after first course of treatment for diarrhea	Stool PCR positive
		500 mg TID	5 days	One year and three months after second course of treatment	N/A
5	Metronidazole	250 mg TID	7 days	After diagnosis	
		250 mg TID	81 days	Three and a half months after first course of treatment for diarrhea until resolution	N/A
6	Metronidazole	500 mg TID	5 days	After diagnosis	
		500 mg BID	120 days	13 months and 10 days after first course of treatment for diarrhea	N/A
7	Metronidazole	250 mg TID	7 days	After diagnosis	
		250 mg TID	7 days	One month after first course of treatment	Stool PCR positive
	Nitazoxanide	500 mg BID	4 days	4 days after second course of treatment	
	Metronidazole	500 mg TID	10 days	One month 10 days after third course of treatment	Stool PCR positive
	Tinidazole	2 g QD	1 day	4 days after fourth course of treatment	N/A

N/A—no stool testing available.
